# Current aboveground live tree carbon stocks and annual net change in forests of conterminous United States

**DOI:** 10.1186/s13021-021-00179-2

**Published:** 2021-05-20

**Authors:** Coeli M. Hoover, James E. Smith

**Affiliations:** USDA Forest Service, Northern Research Station, 271 Mast Road, Durham, NH 03824 USA

**Keywords:** Forest carbon accumulation, Aboveground live tree carbon, Regional and state-level forest carbon stock, Average annual change

## Abstract

**Background:**

With the introduction of the Trillion Trees Initiative and similar programs, forests’ ability to absorb carbon dioxide is increasingly in the spotlight. Many states have mandates to develop climate action plans, of which forest carbon is an important component, and planners need current information on forest carbon stocks and rates of change at relevant spatial scales. To this end, we examine rates of average annual change in live aboveground tree carbon in different forest type groups and provide state-wide and regional summaries of current live tree carbon stock and rates of change for the forests of the conterminous United States. Forest carbon summaries are presented in a format designed to meet the needs of managers, policymakers, and others requiring current estimates of aboveground live tree carbon at state and regional scales.

**Results:**

Regional average aboveground live tree carbon stocks (represented on a per area basis) are generally between 40 and 75 tC/ha but range from 12.8 tC/ha in the Great Plains to 130 tC/ha in the Pacific Northwest West (west-side of Cascades). Regional average annual change in live aboveground tree carbon varies from a low of − 0.18 mtC/ha/y in the Rocky Mountain South to a high value of 1.74 mtC/ha/y in Pacific Northwest West. For individual states, carbon per unit area varies widely, from a low of 11.9 tC/ha in Nevada to a high of 96.4 tC/ha in Washington, with half the states falling between 50 and 75 tC/ha. Rates of average annual change in live aboveground tree carbon vary from a high of 1.82 tC/ha/y in Mississippi to a low of − 0.47 tC/ha/y in Colorado.

**Conclusions:**

Aboveground live tree carbon stocks and rates of average annual change vary by forest type within regions. While softwood forest types currently exhibit a higher rate of increase in the amount of carbon in aboveground live tree biomass, the current standing stock of carbon per unit area does not consistently follow this pattern. For this reason, we recommend computing and considering both measures -standing stock and average annual change—of carbon storage. The relative importance of each component will depend on management and policy objectives and the time frame related to those objectives. Harvesting and natural disturbance also affect forest carbon stock and change and may need to be considered if developing projections of potential carbon storage.

**Supplementary Information:**

The online version contains supplementary material available at 10.1186/s13021-021-00179-2.

## Background

With the introduction of the Trillion Trees Initiative and similar programs, forests’ ability to absorb carbon dioxide is increasingly in the spotlight. More states now have mandates to develop climate action plans, of which forest carbon is an important component. To develop appropriate policies and management strategies, managers, policymakers, landowners, and other practitioners need current information on forest carbon stocks and rates of change at relevant spatial scales (downscaled to the state and regional level). Average carbon values presented on a per area basis can also be used by those practitioners and stakeholders working at the stand, parcel, or small landscape level who do not have access to site-specific data. A set of reference forest carbon stock values for U.S. forests [1] has been cited 406 times, demonstrating the utility of tabular summaries such as those presented here.

Since 1994, the United States has been a party to the United Nations Framework Convention on Climate Change (UNFCCC), which requires the parties to submit annual reports on greenhouse gas sources and sinks [2]. The Intergovernmental Panel on Climate Change (IPCC) is the main scientific body that provides technical guidance to the parties to the UNFCCC on many topics, including reporting. Because of these international reporting obligations (and also various Federal reporting requirements), for global and national-level policy making, much of the available information related to forest carbon sequestration in the United States is summarized and presented according to IPCC guidelines and is not easily translated into estimates useful to managers and policymakers (for example, see [3, 4]). These reports typically provide estimates in terms of carbon dioxide equivalents for total stock or change for a large land area (e.g. regionally or nationally), and use IPCC classifications related to the UNFCCC rather than carbon market or state climate protocols with which managers may be more familiar.

The primary data source for estimates of U.S. forest carbon is the forest inventory of the Forest Inventory and Analysis (FIA) Program of the USDA Forest Service [5]. Previous studies [6–11] have presented estimates of forest carbon, but inventory design and available information as well as biomass equations for most tree species have changed since these analyses [10, 12, 13]. FIA forest inventories transitioned to a nationally consistent annualized remeasurement system starting in the late 1990s, with all conterminous states included by 2011. Estimates of carbon accumulation rates were generally derived from differences in chronological data (overall stocks at two points in time) rather than from re-measured data that are now available from the annualized inventory approach. Most of the past stock-change approaches necessarily conflated effects of growth and land use change due to data limitations. Carbon stock estimates were frequently reported as area-wide stocks for large geographic regions, often without accompanying information on forested area. Many ecological studies that provide estimates of carbon uptake by forests, often at a limited scope, are available. However, these estimates are often developed from flux measurements, process modeling, or a combination of models and data and are focused on quantifying net ecosystem productivity or net primary productivity. While valuable for advancing our understanding of carbon cycling and characterizing the status and trends of a given area, these estimates generally require additional computations or conversions and are of limited use for meeting the information needs of managers, practitioners, and policymakers.

Van Deusen and Heath [14] describe methods that were implemented in an online tool for users to quickly and easily create these estimates of stock specific change (using FIA data) for smaller areas designated by the user, but the tool is not currently available while it is being updated. Customized forest carbon estimates may be generated from FIA data [5], but this process may be challenging for users unfamiliar with FIA data and tools.

Forest carbon sequestration has two major components to consider: stock, or how much carbon is currently stored in a forest; and rate, which is the average annual change in carbon stock. Stock and rate estimates provide different and complementary information; when taken together, they provide a more complete picture of forest carbon, especially in a planning context. While estimates of forest carbon stock provide information on how much carbon is stored in a forest, rate estimates describe how that amount of carbon is changing. For example, average annual change may be used to generate estimates of expected carbon sequestration over the next decade, to assess trends in carbon accumulation, or to compare forest management practices.

Our objective here is not to engage in comprehensive carbon accounting; we focus on live aboveground tree carbon since this pool is large, dynamic, and can be directly influenced by management (live belowground carbon can also be estimated allometrically, and while variable, is generally around 20–25% of aboveground carbon in temperate forests [15, 16]). In addition, these summaries are based directly on field measurements, while other ecosystem carbon pools (not presented here) are obtained from measurements on a more limited set of plots, combined with modeling and interpolation [10]. Our goal is to present current state-wide and regional summaries of aboveground live tree carbon stock and rates of average annual change on forest land remaining forest land for the conterminous United States in an easily used format. We provide this information by “recasting” a portion of the current forest GHG reporting [3] to a scale (state and small region) and scope (forest land remaining forest, expressed on a per hectare basis) to meet the information needs of managers, policymakers, and other practitioners engaged in efforts related to forest-based greenhouse gas mitigation.

## Methods

**Fig. 1:**
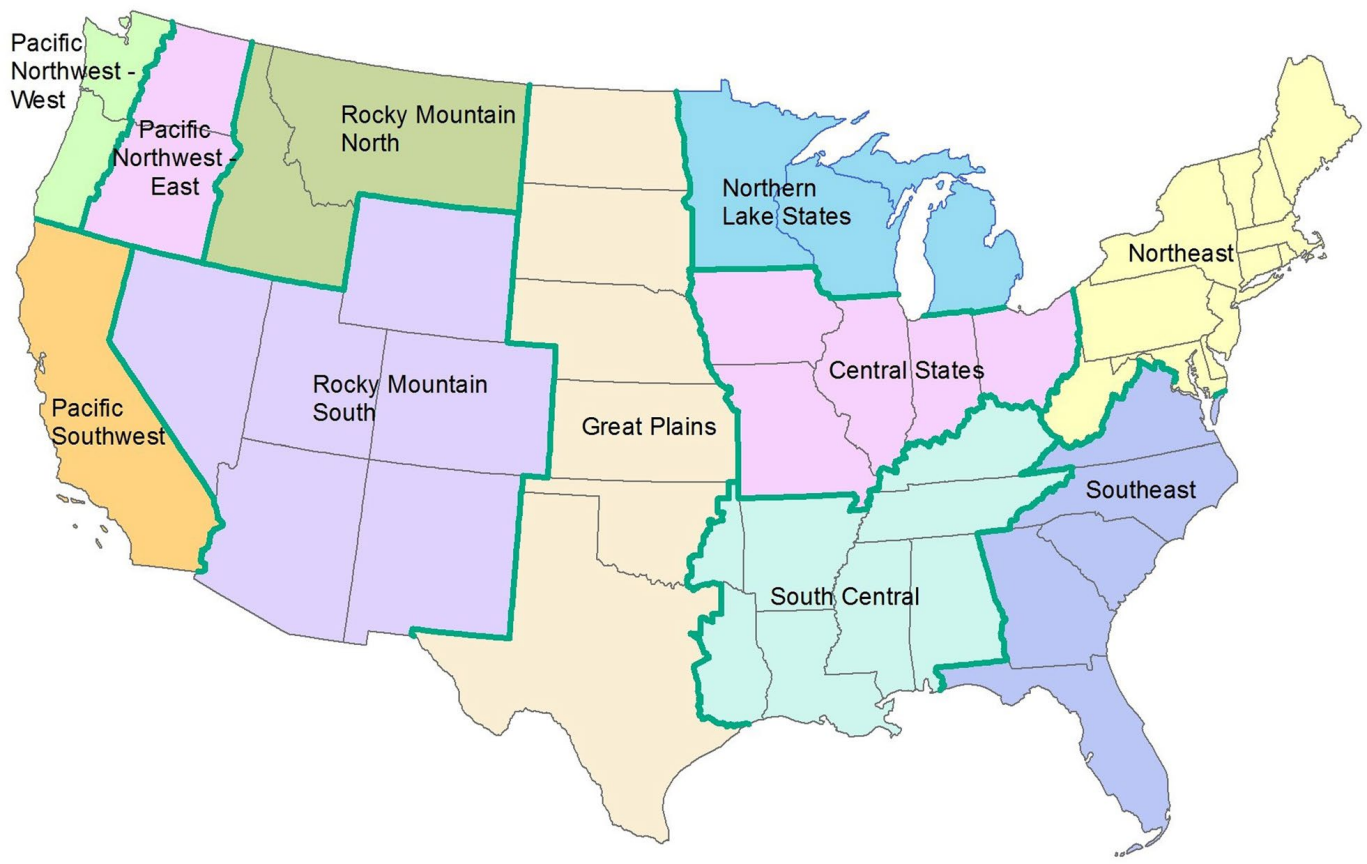
Illustration of geographic regions used for summarizing data

Forest carbon summaries are based on FIA forest inventory data, which were obtained from the publicly available Datamart [5] on 23 June 2020. Forest land of the 48 conterminous states is represented here and classified by state or region as illustrated in Fig. 1 [17]. Stock estimates are taken from the most current survey data available per state, where live tree carbon and forest land are defined as in the inventory database [5]. We determined whole-state and regional carbon stocks for aboveground live trees as the ratio estimates—metric tons carbon per hectare—as described in Bechtold and Patterson [18]. Estimates of carbon stocks may be presented in several formats; we present all estimates as carbon per unit area (carbon density) in order to facilitate comparisons and permit users to generate estimates at a scale that meets their needs.

Stocks of two separate entire inventory surveys are almost inevitably disjoint sets due to some lands moving out of forest use while others change to forest land. Therefore, any such stock change may also include effects of land use change. Because remeasurement data from permanent inventory plots are now widely available, we calculate change in aboveground live tree carbon stock by aggregating change on only those inventory plots that remain forested over the time-1 to time-2 remeasurement interval, which avoids incorporating effects of land use change as in some past stock-change approaches (e.g., [19, 20]). Most states in the conterminous US have a large number of such remeasured continuous-forest plots useful to characterize forest types, age classes, ownerships, and management practices, as well as removals and disturbances. Note that Wyoming and central and western Oklahoma and Texas do not have a sufficient number of remeasurements for inclusion. The changes summarized here are based on plots in the most-recent surveys paired with previous survey measurements on the same permanent inventory plots (i.e., forest plots from prior survey that remained in forest), with an approximately 10-year interval. Specifically, the paired remeasured plots in the western regions (Fig. 1) are from consecutive inventory cycles, which are generally 10 years apart, and the pairs in the East are over two such interval (i.e., a span of two consecutive cycles), which are generally 5 to 7 years each for a total interval of approximately 10 to 14 years.

Paired measurements on plots are included for determining average annual change if the forest condition was greater than 50-percent and not re-mapped (i.e., no change in what was considered forest). We identified disturbance or removals during the remeasurement interval that affect live trees at least 12.7 cm diameter. Condition level disturbance codes (in the inventory database) identified likely disturbance, while tracking the fate of live trees at least 12.7 cm diameter at time-1 identified removals. For this analysis, we separate the classifications of natural disturbances (via the disturbance codes) from removals (including harvest, via individual tree records). Estimates of live aboveground tree carbon density are presented for the forest type groups (as defined by the field typgrpcd in [5]) if the group comprises at least 5% of the forested area in the region. Stock change estimates for those same forest type groups are presented where a minimum of 100 remeasured plots are available for the group. We also summarize by vegetation types, which we set as more aggregate classifications of type groups; these are based on typgrpcd and are: softwood (typgrpcd, 100–390, but not 180); hardwood (typgrpcd, 400–990, but not 970); and woodland (typgrpcd, 180 or 970).

## Results

### Regional carbon stock and change

Regional average aboveground live tree carbon ranges from a high of 130 metric tons per hectare (tC/ha) in the Pacific Northwest West (PWW, refer to Fig. 1) to a low of 12.8 tC/ha in the Great Plains region (GP), with most regions roughly between 40 and 75 tC/ha (Table 1). Carbon density is a function of multiple factors including vegetation type, climate, age class distribution, and disturbance regimes. Carbon densities vary widely by forest type within regions; in the East the highest values are found in the white/red/jack pine, oak/hickory, and beech/birch/maple forests of the Northeast (81.7, 82.8, and 72.6 tC/ha), respectively, with the lowest values in the spruce/fir and aspen/birch forests (29.3 and 31.9 tC/ha) of the Northern Lake States. In the West, carbon densities range from a high of 170.1 tC/ha in Pacific Northwest West hemlock/Sitka spruce forests to a low of 7.8 tC/ha in the woodland hardwoods of Rocky Mountain South (Table 2).

**Table 1:** Average annual change (tC/ha/y) and average carbon stock on a per area basis (tC/ha) for aboveground live tree carbon by region (refer to Fig. 1 for regions)

Region	Avg. Ann. change (tC/ha/y)	Number of paired plots	Avg. C stock (tC/ha)	SEM (tC/ha)
Northeast	0.55	8157	70.9	0.28
Northern Lake States	0.40	8094	43.9	0.23
South Central	0.90	9163	53.7	0.21
Southeast	0.96	7576	59.5	0.30
Central States	0.38	3325	57.7	0.37
Great Plains	0.08	423	11.6	0.09
Rocky Mountain—North	− 0.07	3076	40.2	0.43
Rocky Mountain—South	− 0.18	7797	20.1	0.16
Pacific Northwest—East	0.45	3857	46.1	0.57
Pacific Northwest—West	1.74	3336	130.0	1.23
Pacific Southwest	0.58	2536	76.6	0.98

Note that regional stocks are calculated following Bechtold and Patterson [18] while net annual change is based on a subset of the remeasured forest plots remaining forest (see “Methods”)

*SEM* standard error of the mean

**Table 2:** Average carbon stock per area (tC/ha) for aboveground live tree carbon by region (refer to Fig. 1 for regions) and forest type group

Region	Type group^a^	Average C stock (tC/ha)	SEM
Northeast	White/red/jack pine	81.7	1.27
	Spruce/fir	42.3	0.60
	Oak/hickory	82.8	0.57
	Maple/beech/birch	72.6	0.44
Northern Lake States	White/red/jack pine	47.9	0.85
	Spruce/fir	29.3	0.44
	Oak/hickory	55.7	0.63
	Elm/ash/cottonwood	43.0	0.82
	Maple/beech/birch	59.7	0.54
	Aspen/birch	31.9	0.36
South Central	Loblolly/shortleaf pine	53.7	0.41
	Oak/pine	51.0	0.72
	Oak/hickory	58.4	0.37
	Oak/gum/cypress	68.5	0.93
	Elm/ash/cottonwood	47.4	0.96
Southeast	Longleaf/slash pine	42.5	0.71
	Loblolly/shortleaf pine	57.3	0.55
	Oak/pine	54.6	0.90
	Oak/hickory	69.8	0.57
	Oak/gum/cypress	67.9	1.06
Central States	Oak/hickory	58.4	0.42
	Elm/ash/cottonwood	59.5	1.27
	Maple/beech/birch	69.6	1.68
Great Plains	Oak-hickory	21.9	0.35
Rocky Mountain—North	Douglas-fir	47.7	0.79
	Ponderosa pine	31.5	1.17
	Fir/spruce/mountain hemlock	54.3	1.02
	Lodgepole pine	35.7	0.91
Rocky Mountain—South	Pinyon-juniper	12.4	0.10
	Ponderosa pine	37.3	0.68
	Fir-spruce-mountain hemlock	46.3	0.79
	Aspen-birch	31.9	0.86
	Woodland hardwoods	7.8	0.23
Pacific Northwest—East	Douglas-fir	63.2	1.47
	Ponderosa pine	41.2	0.69
	Fir/spruce/mountain hemlock	75.0	1.85
	Lodgepole pine	31.3	0.97
	Other western softwoods	9.2	0.38
Pacific Northwest—West	Douglas-fir	143.3	1.82
	Fir/spruce/mountain hemlock	131.8	3.94
	Hemlock/Sitka spruce	170.1	3.92
	Alder/maple	83.1	2.54
Pacific Southwest	Ponderosa pine	52.1	2.04
	Fir/spruce/mountain hemlock	113.7	4.18
	Other western softwoods	18.5	1.31
	California mixed conifer	106.8	1.88
	Western oak	47.7	1.14
	Tanoak/laurel	130.8	4.93

*SEM* standard error of the mean

^a^Type groups (within regions) are included if they represent at least 5% of forest within the region based on plot selection used here

**Fig. 2:**
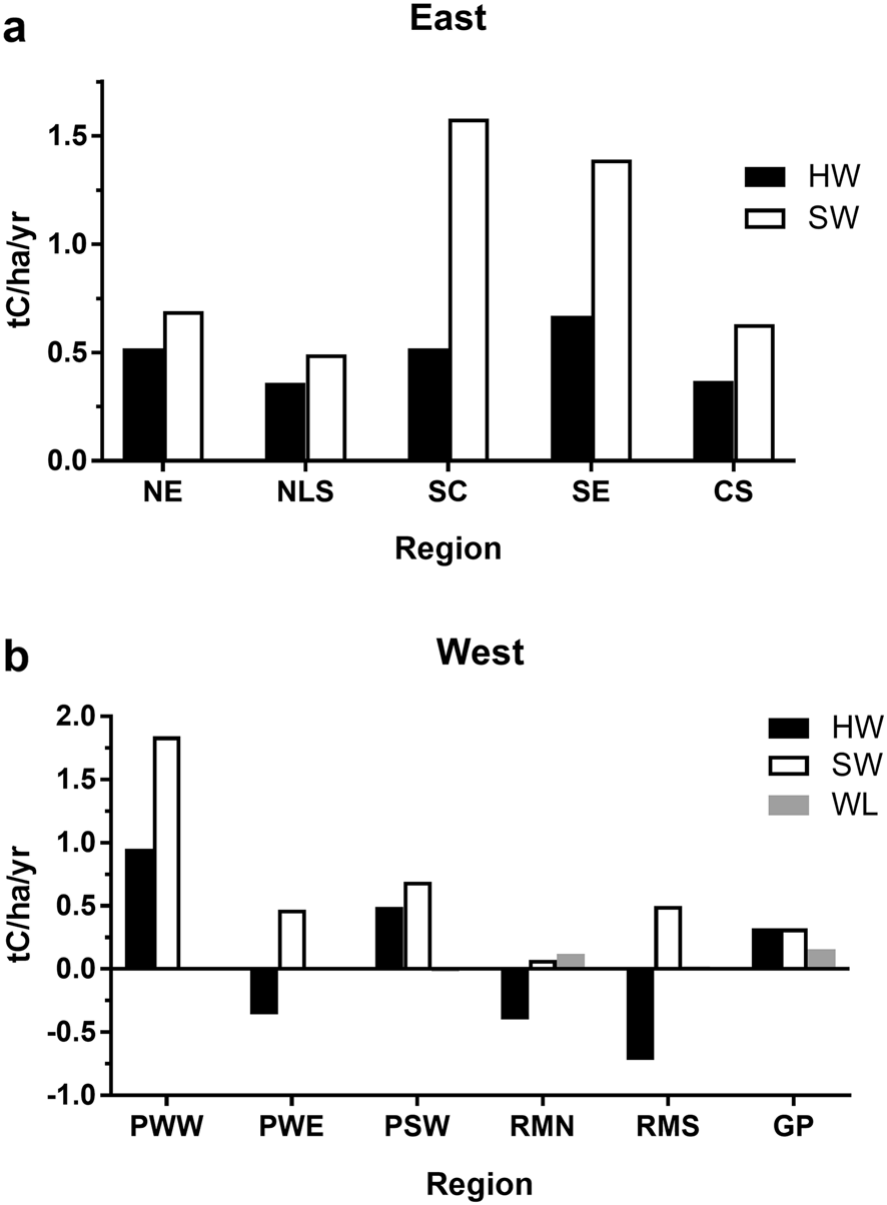
Average annual change in live aboveground tree carbon stocks (tC/ha/y) by vegetation group. **a** Eastern United States. *NE* Northeast, *NLS* Northern Lake States, *SC* South Central, *SE* Southeast, *CS* Central States. **b** Western United States. *PWW* Pacific Northwest West, *PWE* Pacific Northwest East, *PSW* Pacific Southwest, *RMN* Rocky Mountain North, *RMS* Rocky Mountain South, *GP* Great Plains

Average annual change in aboveground live tree carbon varies from a low of − 0.18 tC/ha/y (net carbon loss) in the Rocky Mountain South (RMS) region to a high value of 1.74 tC/ha/y in Pacific Northwest West. Average annual change is variable across the country, although most regions outside the South have values less than 0.6 tC/ha/y (Table 1). Note that while Pacific Northwest West has both the highest carbon density and rate of all the regions, this is not always the case; a region may have a high rate and a lower density, or vice versa. As with carbon density, average annual change is a function of multiple factors, including forest type. In both eastern and western forests, hardwood types accumulate live tree carbon at a slower rate than softwood types (Fig. 2); in western states, live tree carbon in hardwood types is often decreasing (Fig. 2b). Looking more closely at rates by forest type groups, the types with the highest stocks do not necessarily exhibit the highest accumulation rates; for example, the southern pine types have the highest rates in the East (1.08–1.62 tC/ha/y) although carbon densities are higher in the Northeast, and average annual change in Douglas-fir (2.26 tC/ha/y) far exceeds that of hemlock/Sitka spruce (1.03 tC/ha/y) in the Pacific Northwest West region (Table 3).

**Table 3:** Average annual change (tC/ha/y) for aboveground live tree carbon by region (refer to Fig. 1 for regions) and forest type group

Region	Type group^a^	Average annual change (tC/ha/y)	Number of paired plots
Northeast	White/red/jack pine	0.66	429
	Spruce/fir	0.65	973
	Oak/hickory	0.79	2124
	Maple/beech/birch	0.35	3518
Northern Lake States	White/red/jack pine	0.74	762
	Spruce/fir	0.35	1371
	Oak/hickory	0.42	1395
	Elm/ash/cottonwood	0.37	601
	Maple/beech/birch	0.23	1789
	Aspen/birch	0.45	1904
South Central	Loblolly/shortleaf pine	1.62	2972
	Oak/pine	0.64	760
	Oak/hickory	0.43	3744
	Oak/gum/cypress	0.82	814
	Elm/ash/cottonwood	0.66	402
Southeast	Longleaf/slash pine	1.08	813
	Loblolly/shortleaf pine	1.51	2216
	Oak/pine	0.58	766
	Oak/hickory	0.73	2529
	Oak/gum/cypress	0.70	947
Central States	Oak/hickory	0.37	2538
	Elm/ash/cottonwood	0.70	258
	Maple/beech/birch	0.14	191
Great Plains	Oak-hickory	0.44	139
Rocky Mountain—North	Douglas-fir	0.03	1009
	Ponderosa pine	0.34	275
	Fir/spruce/mountain hemlock	0.04	863
	Lodgepole pine	− 0.66	456
Rocky Mountain—South	Pinyon-juniper	0.03	4399
	Ponderosa pine	0.23	598
	Fir-spruce-mountain hemlock	− 0.98	727
	Aspen-birch	− 0.75	504
	Woodland hardwoods	− 0.19	1006
Pacific Northwest—East	Douglas-fir	0.57	744
	Ponderosa pine	0.50	1238
	Fir/spruce/mountain hemlock	0.54	801
	Lodgepole pine	0.19	515
	Other western softwoods	0.04	313
Pacific Northwest—West	Douglas-fir	2.26	1943
	Fir/spruce/mountain hemlock	1.07	467
	Hemlock/Sitka spruce	1.03	460
	Alder/maple	1.36	174
Pacific Southwest	Ponderosa pine	0.71	200
	Fir/spruce/mountain hemlock	0.34	198
	Other western softwoods	0.15	174
	California mixed conifer	0.57	749
	Western oak	0.15	604
	Tanoak/laurel	2.04	144

^a^Type groups (within regions) are included if they represent at least 5% of forest within the region and are represented by at least 100 plots, based on plot selection used here

**Fig. 3:**
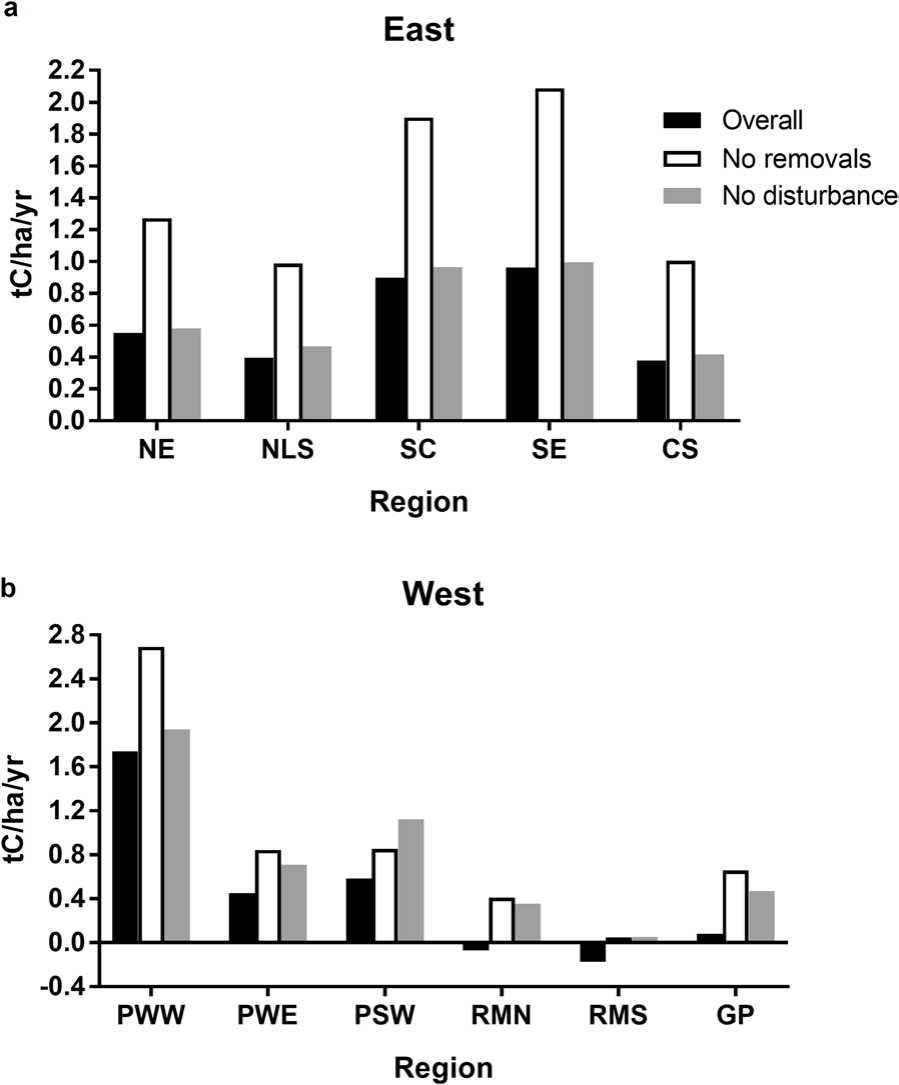
Average annual change in live aboveground tree carbon stocks (tC/ha/y) with all paired plots, with harvested plots excluded, and with naturally disturbed plots excluded. **a** Eastern United States. *NE* Northeast, *NLS* Northern Lake States, *SC* South Central, *SE* Southeast, *CS* Central States. **b** Western United States. *PWW* Pacific Northwest West, *PWE* Pacific Northwest East, *PSW* Pacific Southwest, *RMN* Rocky Mountain North, *RMS* Rocky Mountain South, *GP* Great Plains

Disturbances and removals affect calculations of annual change as presented here. We include an informal analysis of possible regional effects comparing overall regional average annual change with averages based on ‘No removals’ and ‘No disturbance’ (Fig. 3). Here, the second and third bars for each region are summaries from the same records used for ‘Overall’ after removal of plots with identified removals or disturbance over the remeasurement interval, respectively. Note that these forest ecosystem potential changes illustrated here are not intended as comprehensive forest carbon accounting, which tracks wood removed from forests or dead wood remaining in forests.

### State level carbon stock and change

**Fig. 4:**
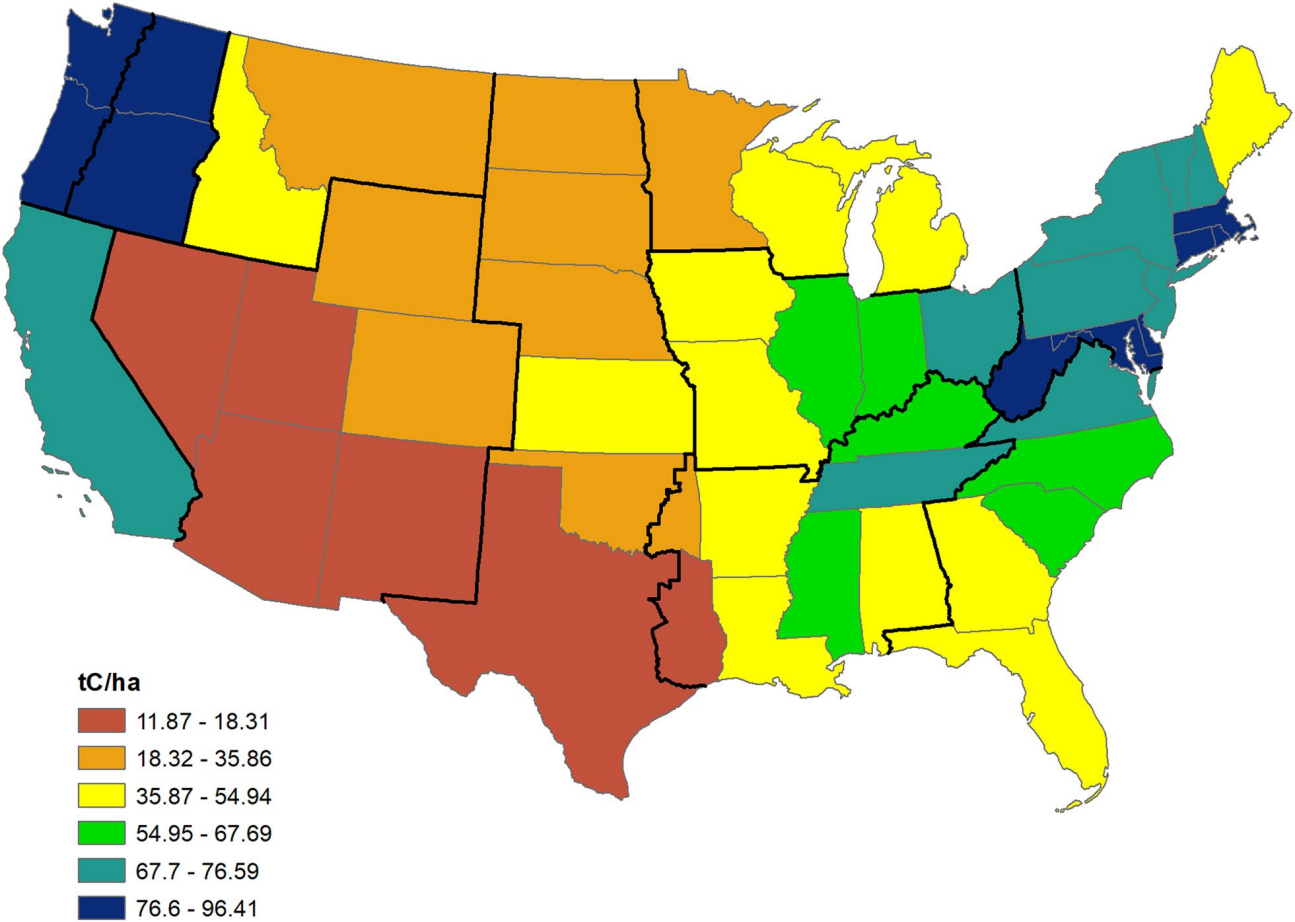
Map of current carbon density (tC/ha) of live tree aboveground biomass. Refer to Additional file 1: Table S1 for sub-state values for states that span more than one region (Texas, Oklahoma, Oregon, Washington)

**Fig. 5:**
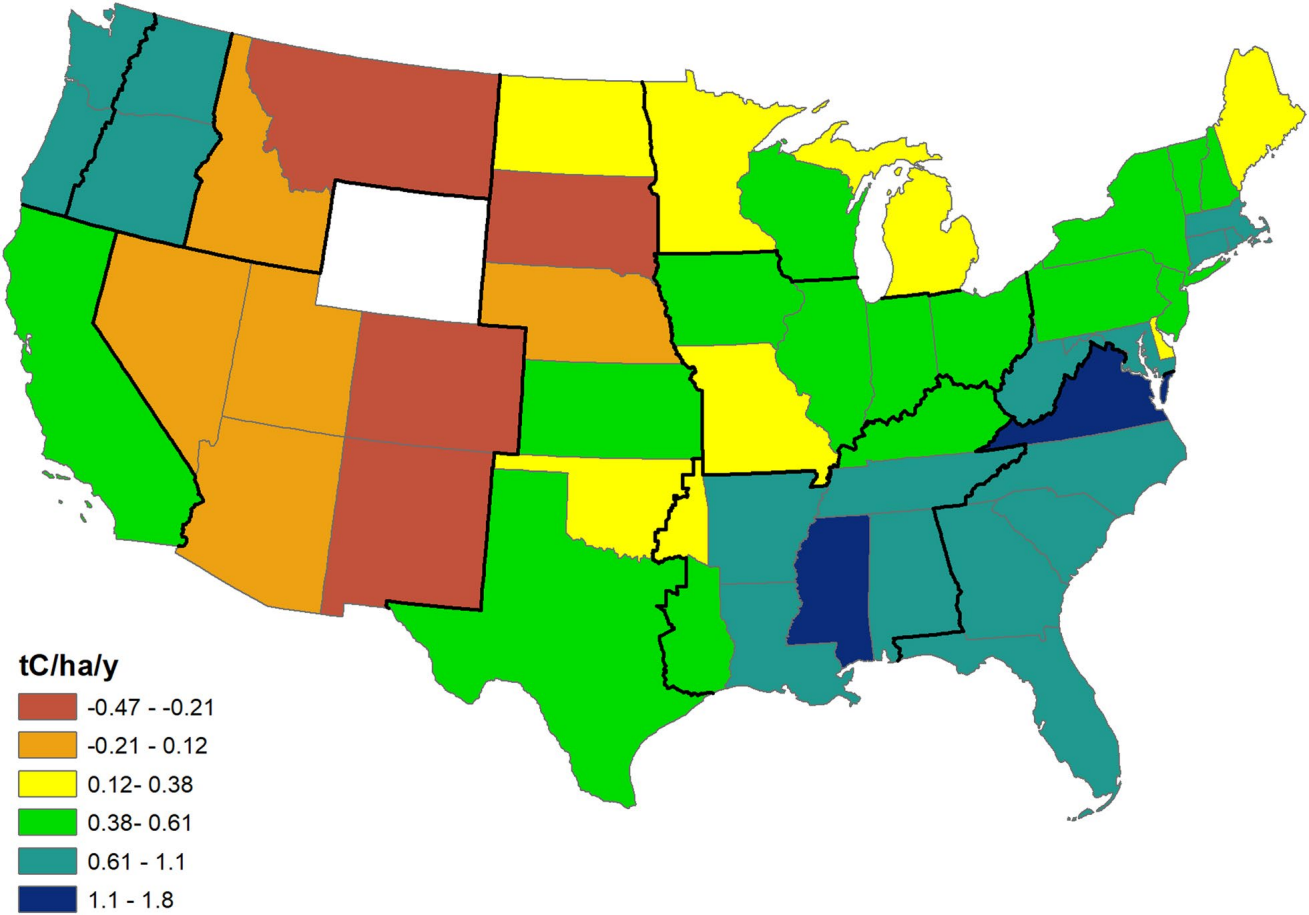
Map of average annual carbon change (tC/ha/y) of live tree aboveground biomass. No value is shown for Wyoming due to a lack of remeasured plots; values for Oklahoma apply only to eastern the eastern portion of the state for the same reason. Refer to Additional file 2: Table S2 for sub-state values for states that span more than one region (Texas, Oklahoma, Oregon, Washington)

Live tree aboveground carbon stock per unit area varies widely, ranging from a low of 11.9 tC/ha in Nevada to a high of 96.4 tC/ha in Washington, with half the states falling between 50 and 75 tC/ha (for state values, see Additional file 1: Table S1). In general, the highest carbon densities are found in forests on the West Coast and in the Northeast, while lowest values are in the Southwest, interior West, and central states (Fig. 4). Rates of average annual change (aboveground live tree carbon) are also variable, ranging from a high of 1.82 tC/ha/y in Mississippi to a low of − 0.47 tC/ha/y in Colorado (see Additional file 2: Table S2 for state rates. Rates are not available for Wyoming and central and western Oklahoma due to a lack of remeasured plots). Generally, the highest rates of aboveground live tree carbon accumulation occur in forests of the Southeast and the Pacific Northwest states, while the lowest rates are in the Southwest and interior West (Fig. 5). Note that as with the regional summary, carbon densities and rates of change may not correspond. As at the regional level, rates often differ between forest types and these differences may be appreciable; in Louisiana, while the statewide rate is 0.73 tC/ha/y, the rate is driven by softwood types, which average 1.18 tC/ha/y while hardwood types accumulate 0.27 tC/ha/y (Additional file 2).

In addition to the summaries presented above, information provided in Additional files 1, 2, 3, 4 and 5 can be used to develop estimates of expected carbon stock and accumulation in hardwood, softwood, and woodland forestland at the state level. As with any estimates summarized at a larger spatial scale, these summary values do not substitute for site-specific assessments, where available.

## Discussion

### Comparisons to existing estimates

Direct comparisons to existing work are somewhat challenging because reports of forest carbon sequestration are often presented for the total forest area or total area of the region under consideration (recalling that our objective is to address the need for current forest carbon information, downscaled and in a format easily useable by managers and policymakers). Time is an additional consideration in the use of such summary data; forest inventories change as do the approaches to estimating and reporting forest carbon. Many published estimates [1, 7, 8] are based on data collected 10–15 years previously, and partially under the periodic inventory system. In the intervening time, not only is newer data available but methods have changed [10], sometimes with appreciable effects. For example, the change from reporting live tree carbon according to Jenkins et al. [15] to the current FIA component ratio method [5] resulted in a substantial decrease in estimated tree carbon stock values [20]; similarly, Hoover and Smith [21] note that three different approaches to estimating carbon in live tree biomass do not always produce equivalent estimates.

Using FIA data and remote sensing to examine post-disturbance growth, Williams et al. [22] determined net ecosystem productivity for regional and forest type group summaries (with several classifications identical to those presented here). The net change in live tree carbon we present has some similarity to net ecosystem productivity, but net ecosystem productivity also includes changes in non-live carbon pools, as well as belowground live carbon. Although slightly different quantities, carbon accumulation rates by type group and region were generally comparable (see Table 2 of Williams et al. [23] in comparison with Table 3 here). Smith et al. [20] also examined FIA-based forest carbon stock and change with a focus on federal forest lands (although all ownerships were summarized) using the FIA regions North, South, Rocky Mountain, and Pacific Coast. Average aboveground live tree carbon density on United States’ federal forest lands across all ownerships was highest in the Pacific Coast region, which includes Pacific Northwest West, Pacific Northwest East, and Pacific Southwest; this agrees with our results (recalling that Pacific Northwest West and Pacific Southwest had the highest carbon densities). They also found the lowest carbon densities in the Rocky Mountain region, which agrees with our findings. Earlier estimates of forest carbon stock in the National Forest System [19] summarized by National Forest region generally agree, with highest aboveground live tree carbon densities in forests of southeast coastal Alaska (not considered here), and the Pacific Northwest and Pacific Southwest regions of the National Forest System. Heath et al. [19] also report the lowest carbon densities in the Southwestern and Intermountain regions (generally corresponding to Rocky Mountain South although not an exact match). Harris et al. [24] used a combination of FIA data and remote sensing methods to examine forest carbon stock and change across the US with a focus on disturbance; mapped carbon densities agree with values reported here. Similarly, FIA based state level forest inventory reports that summarize carbon stocks [25, 26] present estimates consistent with those provided here, which is expected because of the common data sources.

State-level estimates of carbon density and change are available in several reports; as mentioned previously, these numbers require additional conversions in order to be compared to the present study. While recent US EPA estimates include state-level estimates of total forest carbon [3, 4], they do not include forest areas, which are necessary to develop these summaries. While state level forest area and carbon summaries are included in some previous EPA reports [27], it does not separately report live tree carbon; USDA [28] is based on the same data as US EPA [27] and does report comparable live tree carbon and forest area per state. However, stock change reported is based on the older stock-change approach, which does not separately identify or exclude effects of land-use change. Comparing five states from different regions (Maine, Wisconsin, Alabama, Colorado, and Washington) we find generally good agreement in carbon density between Additional file 1: Table S1 here and [28], which includes belowground live tree carbon. The largest differences (16.3 and 12.8 tC/ha) are in Washington and Colorado, respectively, while the smallest differences are in Alabama and Wisconsin (2.9 and 5.5 tC/ha).

Note that any estimates of forest carbon stocks or rates of change developed at a state or regional level are average values and should not be expected to represent conditions on any specific parcel, since many factors influence stand growth. However, in the absence of site specific data, reference values serve the function of providing reasonable values of carbon density and rate of change (in this case, for the aboveground portion of live trees) that may be expected in a given forest type group or region. Rates of average annual change presented here represent an approximate period from 2007 to 2016, plus or minus a few years, depending on the state. This interval should provide reasonable values of forest change over the near term, although rates are expected to change over time as influenced by many factors including weather, disturbance, and changes in age class distribution.

### Forest remaining forest

Carbon accumulation rates in the literature are rarely reported on a per area basis and are commonly presented as total carbon mass for stock (or change) for large geographic regions [3, 4], which is not clearly linked to area or area change, leading to challenges when attempting to use the estimates as reference values. The principal reason for this is that these reports are oriented toward whole-country reporting of greenhouse gas inventories, and change in carbon density on forestland does not sum to total stock change for the country (the reporting focus). This is because the interval generally includes a portion of non-forestland becoming forest and forest becoming non-forest, see the discussion of accounting for greenhouse gas emissions and land use change in Chap. 6 of US EPA [3]. The recent-year accumulation of remeasurement data on the permanent FIA annualized inventory plots as well as increased information on land use change as it affects forests and forest carbon is changing approaches to reporting [3, 4]; our approach to determining annual change is consistent with this direction in reporting.

Many of the past reports (as reviewed above) were unable to separate effects of land use. Therefore, prior to FIA implementing an annual inventory system and the availability of data from remeasured plots, changes in aboveground live tree carbon stocks were generally computed as the difference in carbon stock measurements at two points in time, which necessarily includes the effects of land transitioning into and out of forest. Effects of changing area of forest land on carbon change estimates can be low, but the difficulty is that they were unknown. For example, a comparison of mostly similar change summaries per state (Additional file 2: Table S2) relative to state summaries in USDA [28] shows generally good agreement for many states, with the largest difference approximately 0.25 tC/ha/yr, for both Washington and Colorado. No pattern of consistently higher or lower estimates of average annual change were noted between the two approaches. As an extension of this comparison between the remeasured-plot (here) versus stock-change (e.g., Smith et al. [29]) approaches, we calculated change following both methods for our dataset. The results are in Additional file 5: Table S5, which also shows variable levels of agreement with no pattern of extreme differences. The current estimates of carbon accumulation rates reported here are based on a subset of all available plots; those that have been remeasured and are majority one forest condition class. As such, change is a slightly different quantity than is required with whole-state or national-level reporting—here, change in live tree carbon on forested lands is the sum of survivor growth, in-growth, mortality, and removals.

While land-use change is an important component of forest carbon estimates, it is difficult to link land use change to quantities of current and expected future carbon stocks that are needed for applications such as state climate action plans or forest carbon project feasibility assessments. For this reason, when assessing change we focus on remeasured plots on forest land remaining in forest. One of our main objectives is to provide current estimates of aboveground live tree carbon density and average annual change in a format and at a level of aggregation useful for managers and policymakers seeking reference values to aid in understanding the current state of forest carbon or developing forest carbon plans.

### Role of harvest and natural disturbance

While forest carbon density is increasing in most regions, in both Rocky Mountain regions carbon in live tree aboveground biomass is decreasing, and carbon densities in the Southeast and South Central regions are lower than those in the Northeast (Table 1). A possible explanation is harvesting and natural disturbance; several studies [22, 24, 30, 31] estimate the effects of harvest and natural disturbance on United States forest carbon stocks. Effects vary by region, with the largest impacts generally in the South and West. When plots with removals or disturbance activity during our study period are excluded, mean aboveground live tree carbon accumulation rates of the remaining records can increase considerably, with the smaller effect from disturbance. Williams et al. [22] identified this result as reflecting harvesting practices and regional climates with the greatest effects in the Southeast, South Central, and Pacific Northwest regions. This is evident in our results for the East, in harvest effects in South Central and Southeast regions (Fig. 3a); natural disturbance has a small effect. In the West, harvesting has a substantial impact in the Pacific Northwest West region (and is noticeable in the Great Plains), but natural disturbance appears to have decreased live tree carbon accumulation rates in all regions, most clearly in the Rocky Mountain North, Rocky Mountain South, and Great Plains regions (Fig. 3b). Note that while harvesting generally results in higher growth rates when a stand is regenerated, this effect is not illustrated in Fig. 3, which simply reflects average effect on all forest at all stages, without disturbance or removal effects.

### Scope of this analysis

The amount of live tree biomass on the landscape, and the change in that quantity, are a function of many variables, including site productivity, species mix, age class distribution, and ownership. In this work, our primary objective is to provide estimates in a format easily used by those requiring current information on the state of aboveground live tree carbon. Past work [21] has examined the role of site productivity using the productivity classification in the FIA database, and future work will focus on the effect of age class distribution, which has pronounced effects on estimates of live tree carbon stock and change. In order to maintain a sufficient number of plots in each classification, especially at the state level, estimates for forest type by age class are not feasible using the current dataset.

Note that we are not making region-to-region comparisons at the scale of the summaries provided here because forest biomes, climate, and land use vary across the country. Quantifying or testing differences among regions is of limited use without reducing an analysis to a relatively limited number of factors. Even reducing summaries to forest type group has limited value as a basis for comparing across regions because most groups summarize several forest types, which can be regionally specific. For example, the white/red/jack pine groups in Northeast and Northern Lake States are predominantly different types; in the Northeast 82% of stands are Eastern white pine or Eastern hemlock stands, while in Northern Lake States these pines are 72% jack or red pine. So, largely different pine forests are represented in the two regions. Regional differences in type groups such as seen in Tables 2 and 3 have been reported by Smith et al. [1] and Williams et al. [24].

These estimates are developed from current FIA data and do not include a modeling component but reflect current live tree carbon densities and average annual change in forests including the effects of management, natural disturbance, and harvesting. Our choices to limit forest carbon estimates to aboveground live tree carbon, include only forest-remaining-forest (factoring out land use change), and set political bounds rather than ecological divisions of the land base are all aimed at providing a more easily used reference for state level analysis/planning. The data summarized here are essentially the same as what goes into the whole-U.S. forest carbon reporting in US EPA [3] except that scale and the carbon pool focus are narrowed considerably. It is useful to note that while the summarized data are derived from the same source, the values here are not readily summed back to the whole-country reported values; this is primarily because the net change we present is strictly limited to forest-remaining-forest change over generally longer intervals.

## Conclusions

Current aboveground live tree carbon density varies widely among the regions and states, with the highest carbon densities found in the Pacific Northwest West, Pacific Southwest, and Northeast regions, and the lowest in the Great Plains and Rocky Mountain South. At the state level, states in the Pacific Northwest and Northeast had the highest carbon stock per hectare while densities were lowest in Southwestern states. Average annual change in live aboveground tree carbon is also variable; highest rates are found in the Pacific Northwest West, Southeast, and Southern regions, while both Rocky Mountain regions exhibited negative accumulation rates. Carbon density and average annual change also vary by forest type within regions, with higher rates in softwood forest types; carbon density did not consistently follow this pattern. Note that the regional results are not identical for carbon stock (expressed as density) and rate (average annual change per unit area). For this reason, we recommend computing and considering both measures of carbon sequestration; the relative weight given to each will depend on management and policy objectives. Finally, harvesting and natural disturbance also affect the forest carbon sink and may need to be included when developing projections of future carbon storage potential or plans related to maintaining or enhancing the forest carbon sink.

## Supplementary Information

**Additional file 1: Table S1.** Average current carbon stock by state (aboveground live tree carbon) on a per area basis (tC/ha).

**Additional file 2: Table S2.** Rate of average annual change (live aboveground tree carbon only) by state and vegetation class (tC/ha/y).

**Additional file 3: Table S3.** Carbon stock (live aboveground tree carbon only) on a per area basis by state and vegetation class (tC/ha).

**Additional file 4: Table S4.** Forested area by state and vegetation class.

**Additional file 5: Table S5.** Rates of average annual (aboveground live tree) change by state as calculated from two approaches.

## Data Availability

The dataset supporting the conclusions of this article is available in the FIA Data Mart [https://apps.fs.usda.gov/fia/datamart/datamart.html]. Note that the database is updated routinely.
