# Supplementary material for: Current aboveground live tree carbon stocks and annual net change in forests of conterminous United States
Source: Carbon Balance Manag. 2021 May 20;16:17. doi: 10.1186/s13021-021-00179-2 (PMC8138985; doi:10.1186/s13021-021-00179-2)
Supplement: Supplementary file 1 — Additional file 1: Table S1. Average current carbon stock by state (aboveground live tree carbon) on a per area basis (tC/ha). [file 13021_2021_179_MOESM1_ESM.docx]

Table S1. Average current carbon stock by state (aboveground live tree carbon) on a per area basis (tonnes C/ha). SEM; standard error of the mean. Note that for states that cross regional boundaries, estimates are presented for the entire state as well as for the portion in each region.

| **State** | **C stock** | **SEM** |  | **State** | **C stock** | **SEM** |
| --- | --- | --- | --- | --- | --- | --- |
|  | (tC/ha) | (tC/ha) |  |  | (tC/ha) | (tC/ha) |
| Alabama | 53.7 | 0.52 |  | Oklahoma | 27.8 | 0.32 |
| Arizona | 16.8 | 0.35 |  | Oklahoma (Great Plains) | 21.6 | 0.54 |
| Arkansas | 54.0 | 0.57 |  | Oklahoma (South Central) | 34.8 | 0.70 |
| California | 76.6 | 0.98 |  | Oregon | 85.9 | 0.88 |
| Colorado | 29.8 | 0.44 |  | Oregon (West) | 128.6 | 1.57 |
| Connecticut | 87.3 | 2.07 |  | Oregon (East) | 40.5 | 0.62 |
| Delaware | 84.2 | 3.72 |  | Pennsylvania | 76.2 | 0.69 |
| Florida | 43.8 | 0.66 |  | Rhode Island | 81.1 | 2.89 |
| Georgia | 54.9 | 0.54 |  | South Carolina | 58.7 | 0.80 |
| Idaho | 46.2 | 0.74 |  | South Dakota | 25.9 | 0.96 |
| Illinois | 59.6 | 1.01 |  | Tennessee | 69.3 | 0.73 |
| Indiana | 66.7 | 1.11 |  | Texas | 15.4 | 0.12 |
| Iowa | 50.0 | 1.36 |  | Texas (Great Plains) | 8.8 | 0.11 |
| Kansas | 41.4 | 1.29 |  | Texas (South Central) | 42.7 | 0.64 |
| Kentucky | 65.5 | 0.69 |  | Utah | 18.3 | 0.34 |
| Louisiana | 51.7 | 0.70 |  | Vermont | 75.3 | 1.09 |
| Maine | 47.7 | 0.44 |  | Virginia | 74.1 | 0.73 |
| Maryland | 89.6 | 2.22 |  | Washington | 96.4 | 1.17 |
| Massachusetts | 86.1 | 1.53 |  | Washington (West) | 131.8 | 1.95 |
| Michigan | 50.3 | 0.49 |  | Washington (East) | 54.1 | 1.05 |
| Minnesota | 34.4 | 0.29 |  | West Virginia | 80.7 | 0.84 |
| Mississippi | 61.0 | 0.63 |  | Wisconsin | 46.4 | 0.35 |
| Missouri | 49.6 | 0.40 |  | Wyoming | 29.0 | 0.72 |
| Montana | 35.1 | 0.49 |  |  |  |  |
| Nebraska | 35.9 | 1.98 |  |  |  |  |
| Nevada | 11.9 | 0.27 |  |  |  |  |
| New Hampshire | 72.4 | 1.12 |  |  |  |  |
| New Jersey | 69.7 | 1.50 |  |  |  |  |
| New Mexico | 14.6 | 0.28 |  |  |  |  |
| New York | 73.3 | 0.63 |  |  |  |  |
| North Carolina | 67.7 | 0.72 |  |  |  |  |
| North Dakota | 27.8 | 1.78 |  |  |  |  |
| Ohio | 70.0 | 1.03 |  |  |  |  |
