# Supplementary material for: Current aboveground live tree carbon stocks and annual net change in forests of conterminous United States
Source: Carbon Balance Manag. 2021 May 20;16:17. doi: 10.1186/s13021-021-00179-2 (PMC8138985; doi:10.1186/s13021-021-00179-2)
Supplement: Supplementary file 2 — Additional file 2: Table S2. Rate of average annual change (live aboveground tree carbon only) by state and vegetation class (tC/ha/y). [file 13021_2021_179_MOESM2_ESM.docx]

Table S2. Carbon accumulation rates (live aboveground tree carbon only) by state and vegetation class (tC/ha/y). N = of plots on which the estimate is based. Blank cells indicate no plots classified as woodland. Hardwood, softwood, and woodland classifications based on forest type groups, as described in Methods. Note that for states that cross regional boundaries, estimates are presented for the entire state as well as for the portion in each region. Wyoming as well as the Great Plains portions of Oklahoma and Texas are not included because of a lack of remeasured plots.

| **State** | **Overall** | **N** | **Hardwood** | **N** | **Softwood** | **N** | **Woodland** | **N** |
| --- | --- | --- | --- | --- | --- | --- | --- | --- |
|  | (tC/ha/y) |  | (tC/ha/y) |  | (tC/ha/y) |  | (tC/ha/y) |  |
| Alabama | 0.94 | 1917 | 0.53 | 941 | 1.33 | 976 |  |  |
| Arizona | -0.01 | 1995 | -1.18 | 25 | 0.14 | 347 | -0.03 | 1623 |
| Arkansas | 0.76 | 1823 | 0.43 | 1198 | 1.38 | 624 |  |  |
| California | 0.58 | 2536 | 0.49 | 794 | 0.69 | 1587 | -0.02 | 155 |
| Colorado | -0.47 | 1922 | -0.76 | 289 | -0.78 | 899 | 0.01 | 734 |
| Connecticut | 0.86 | 146 | 0.86 | 145 | 1.01 | 1 |  |  |
| Delaware | 0.27 | 48 | -0.23 | 38 | 2.18 | 10 |  |  |
| Florida | 0.73 | 1148 | 0.56 | 612 | 0.93 | 536 |  |  |
| Georgia | 0.84 | 2144 | 0.34 | 1092 | 1.37 | 1052 |  |  |
| Idaho | 0.12 | 1295 | 0.04 | 50 | 0.12 | 1203 | 0.20 | 42 |
| Illinois | 0.51 | 404 | 0.51 | 393 | 0.64 | 11 |  |  |
| Indiana | 0.51 | 453 | 0.52 | 438 | 0.38 | 15 |  |  |
| Iowa | 0.55 | 201 | 0.54 | 199 | 1.08 | 2 |  |  |
| Kansas | 0.57 | 118 | 0.57 | 116 | 0.68 | 2 |  |  |
| Kentucky | 0.61 | 1027 | 0.60 | 1006 | 1.12 | 21 |  |  |
| Louisiana | 0.73 | 308 | 0.27 | 154 | 1.18 | 154 |  |  |
| Maine | 0.38 | 2241 | 0.14 | 1308 | 0.72 | 932 |  |  |
| Maryland | 0.83 | 136 | 0.61 | 110 | 1.77 | 26 |  |  |
| Massachusetts | 0.83 | 242 | 0.79 | 204 | 1.03 | 38 |  |  |
| Michigan | 0.38 | 2134 | 0.31 | 1537 | 0.55 | 597 |  |  |
| Minnesota | 0.34 | 2861 | 0.31 | 1889 | 0.39 | 972 |  |  |
| Mississippi | 1.82 | 1450 | 1.00 | 711 | 2.60 | 739 |  |  |
| Missouri | 0.26 | 1600 | 0.25 | 1528 | 0.50 | 72 |  |  |
| Montana | -0.21 | 1781 | -0.82 | 52 | -0.20 | 1652 | 0.08 | 77 |
| Nebraska | 0.08 | 77 | 0.10 | 54 | -0.01 | 22 | 0.76 | 1 |
| Nevada | 0.03 | 734 | -0.26 | 15 | -0.57 | 28 | 0.06 | 691 |
| New Hampshire | 0.59 | 529 | 0.61 | 412 | 0.50 | 117 |  |  |
| New Jersey | 0.57 | 173 | 0.53 | 125 | 0.65 | 48 |  |  |
| New Mexico | -0.24 | 1068 | -3.02 | 21 | -0.33 | 243 | -0.15 | 804 |
| New York | 0.55 | 1836 | 0.55 | 1619 | 0.56 | 217 |  |  |
| North Carolina | 1.03 | 1483 | 0.79 | 938 | 1.45 | 545 |  |  |
| North Dakota | 0.31 | 55 | 0.33 | 50 |  |  | 0.11 | 5 |
| Ohio | 0.44 | 667 | 0.42 | 653 | 1.47 | 14 |  |  |
| Oklahoma (S. Central) | 0.32 | 357 | 0.06 | 261 | 1.02 | 96 |  |  |
| Oregon | 1.07 | 4776 | 0.84 | 299 | 1.09 | 4477 |  |  |
| Oregon (West) | 1.66 | 2080 | 0.80 | 270 | 1.79 | 1810 |  |  |
| Oregon (East) | 0.47 | 2696 | -0.08 | 29 | 0.47 | 2667 |  |  |
| Pennsylvania | 0.57 | 1540 | 0.59 | 1487 | 0.14 | 53 |  |  |
| Rhode Island | 0.84 | 51 | 0.85 | 43 | 0.73 | 8 |  |  |
| South Carolina | 0.87 | 1165 | 0.53 | 551 | 1.17 | 614 |  |  |
| South Dakota | -0.32 | 173 | -0.15 | 36 | -0.39 | 131 | 0.10 | 6 |
| Tennessee | 0.75 | 1210 | 0.68 | 1103 | 1.41 | 107 |  |  |
| Texas (S. Central) | 0.52 | 1071 | -0.17 | 504 | 1.14 | 567 |  |  |
| Utah | -0.09 | 2078 | -0.33 | 174 | -0.54 | 351 | 0.04 | 1553 |
| Vermont | 0.61 | 1636 | 0.61 | 461 | 0.59 | 83 |  |  |
| Virginia | 1.28 | 544 | 0.97 | 1313 | 2.56 | 323 |  |  |
| Washington | 1.00 | 2417 | 0.56 | 141 | 1.03 | 2277 |  |  |
| Washington (West) | 1.66 | 1256 | 0.95 | 104 | 1.72 | 1152 |  |  |
| Washington (East) | 0.21 | 1161 | -0.57 | 37 | 0.23 | 1124 |  |  |
| West Virginia | 0.77 | 671 | 0.76 | 657 | 0.87 | 14 |  |  |
| Wisconsin | 0.46 | 3099 | 0.43 | 2518 | 0.61 | 581 |  |  |
