# Supplementary material for: Current aboveground live tree carbon stocks and annual net change in forests of conterminous United States
Source: Carbon Balance Manag. 2021 May 20;16:17. doi: 10.1186/s13021-021-00179-2 (PMC8138985; doi:10.1186/s13021-021-00179-2)
Supplement: Supplementary file 3 — Additional file 3: Table S3. Carbon stock (live aboveground tree carbon only) on a per area basis by state and vegetation class (tC/ha). [file 13021_2021_179_MOESM3_ESM.docx]

Table S3. Carbon stock (live aboveground tree carbon only) by state and vegetation class (tC/ha). SEM; standard error of the mean. Blank cells indicate no plots classified as woodland. Hardwood, softwood, and woodland classifications based on forest type groups, as described in Methods. Note that for states that cross regional boundaries, estimates are presented for the entire state as well as for the portion in each region.

| **State** | **Hardwood** | **SEM** | **Softwood** | **SEM** | **Woodland** | **SEM** |
| --- | --- | --- | --- | --- | --- | --- |
|  | (tC/ha) | (tC/ha) | (tC/ha) | (tC/ha) | (tC/ha) | (tC/ha) |
| Alabama | 57.0 | 0.77 | 50.5 | 0.71 |  |  |
| Arizona | 33.4 | 6.61 | 47.8 | 1.33 | 11.4 | 0.22 |
| Arkansas | 54.8 | 0.72 | 53.6 | 0.96 | 36.2 | 5.44 |
| California | 62.3 | 1.39 | 97.1 | 1.55 | 9.8 | 0.59 |
| Colorado | 36.1 | 1.21 | 44.3 | 0.73 | 13.9 | 0.28 |
| Connecticut | 88.4 | 2.11 | 87.9 | 13.75 |  |  |
| Delaware | 87.2 | 4.23 | 73.7 | 6.71 |  |  |
| Florida | 49.6 | 1.05 | 39.9 | 0.77 |  |  |
| Georgia | 58.7 | 0.81 | 52.1 | 0.74 |  |  |
| Idaho | 20.8 | 1.70 | 53.7 | 0.83 | 11.8 | 0.91 |
| Illinois | 59.8 | 1.02 | 71.6 | 8.20 |  |  |
| Indiana | 67.5 | 1.12 | 46.3 | 4.84 |  |  |
| Iowa | 51.2 | 1.38 | 19.9 | 3.48 |  |  |
| Kansas | 43.7 | 1.34 | 15.7 | 2.40 |  |  |
| Kentucky | 66.4 | 0.70 | 44.3 | 3.37 |  |  |
| Louisiana | 53.4 | 0.99 | 50.6 | 1.01 |  |  |
| Maine | 49.6 | 0.64 | 45.3 | 0.68 |  |  |
| Maryland | 94.8 | 2.40 | 67.7 | 4.96 |  |  |
| Massachusetts | 85.3 | 1.59 | 92.4 | 4.44 |  |  |
| Michigan | 53.5 | 0.59 | 42.3 | 0.85 |  |  |
| Minnesota | 37.7 | 0.37 | 28.2 | 0.52 |  |  |
| Mississippi | 61.0 | 0.91 | 63.3 | 0.88 |  |  |
| Missouri | 50.2 | 0.40 | 39.9 | 2.10 |  |  |
| Montana | 17.3 | 1.83 | 40.9 | 0.55 | 7.6 | 0.41 |
| Nebraska | 46.9 | 2.85 | 21.9 | 1.99 | 24.8 | 0.84 |
| Nevada | 13.2 | 1.99 | 39.5 | 3.57 | 11.5 | 0.20 |
| New Hampshire | 73.3 | 1.30 | 70.3 | 2.45 |  |  |
| New Jersey | 79.3 | 1.78 | 44.4 | 1.57 |  |  |
| New Mexico | 36.1 | 2.98 | 41.1 | 0.96 | 8.6 | 0.16 |
| New York | 74.9 | 0.68 | 68.4 | 1.79 |  |  |
| North Carolina | 72.9 | 0.93 | 59.7 | 1.12 |  |  |
| North Dakota | 31.1 | 2.01 | 6.1 | 0.00 | 11.5 | 1.64 |
| Ohio | 70.8 | 1.04 | 59.0 | 5.65 |  |  |
| Oklahoma | 29.5 | 0.51 | 31.1 | 1.33 | 5.3 | 0.98 |
| Oklahoma (Great Plains) | 25.4 | 0.63 | 12.0 | 1.25 | 5.3 | 0.98 |
| Oklahoma (South Central) | 34.5 | 0.82 | 38.0 | 1.56 |  |  |
| Oregon | 72.7 | 2.60 | 91.3 | 0.99 | 5.4 | 2.20 |
| Oregon (West) | 77.9 | 2.76 | 143.3 | 1.84 |  |  |
| Oregon (East) | 23.4 | 3.19 | 43.2 | 0.65 | 5.4 | 2.20 |
| Pennsylvania | 77.2 | 0.70 | 63.5 | 3.05 |  |  |
| Rhode Island | 81.7 | 3.00 | 83.1 | 8.67 |  |  |
| South Carolina | 63.0 | 1.24 | 55.5 | 1.02 |  |  |
| South Dakota | 30.0 | 2.24 | 28.9 | 1.16 | 13.7 | 2.06 |
| Tennessee | 71.4 | 0.77 | 53.4 | 2.00 |  |  |
| Texas | 24.8 | 0.38 | 47.6 | 0.97 | 5.7 | 0.09 |
| Texas (Great Plains) | 18.3 | 0.34 | 21.0 | 2.40 | 5.7 | 0.09 |
| Texas (South Central) | 38.6 | 0.84 | 48.7 | 1.00 |  |  |
| Utah | 27.7 | 1.49 | 37.7 | 1.27 | 13.7 | 0.23 |
| Vermont | 75.7 | 1.18 | 74.3 | 3.05 |  |  |
| Virginia | 76.0 | 0.81 | 68.5 | 1.68 |  |  |
| Washington | 69.4 | 2.82 | 104.5 | 1.33 |  |  |
| Washington (West) | 79.4 | 3.25 | 144.1 | 2.27 |  |  |
| Washington (East) | 31.7 | 3.41 | 59.6 | 1.12 |  |  |
| West Virginia | 81.8 | 0.85 | 49.5 | 3.39 |  |  |
| Wisconsin | 48.3 | 0.40 | 40.4 | 0.82 |  |  |
| Wyoming | 20.1 | 1.89 | 35.9 | 0.86 | 9.1 | 0.75 |
