# Supplementary material for: Current aboveground live tree carbon stocks and annual net change in forests of conterminous United States
Source: Carbon Balance Manag. 2021 May 20;16:17. doi: 10.1186/s13021-021-00179-2 (PMC8138985; doi:10.1186/s13021-021-00179-2)
Supplement: Supplementary file 4 — Additional file 4: Table S4. Forested area by state and vegetation class. [file 13021_2021_179_MOESM4_ESM.docx]

Table S4. Forested area by state and vegetation class. % = percentage of total forestland in that vegetation class. Hardwood, softwood, and woodland classifications based on forest type groups, as described in Methods. Note that percentages will not sum to 100 because nonstocked forest land is not included. For states that cross regional boundaries, estimates are presented for the entire state as well as for the portion in each region.

| **State** | **Forest Area** | **Hardwood** | **%** | **Softwood** | **%** | **Woodland** | **%** |
| --- | --- | --- | --- | --- | --- | --- | --- |
|  | (thousand ha) | (thousand ha) |  | (thousand ha) |  | (thousand ha) |  |
| Alabama | 9,350 | 501 | 53.6 | 4,280 | 45.8 |  |  |
| Arizona | 7,478 | 95 | 1.3 | 1,151 | 15.4 | 5,889 | 78.8 |
| Arkansas | 7,659 | 5,010 | 65.4 | 2,584 | 33.7 | 8 | 0.1 |
| California | 12,819 | 4,642 | 36.2 | 7,052 | 55.0 | 678 | 5.3 |
| Colorado | 9,283 | 1,443 | 15.6 | 3,940 | 42.4 | 3,592 | 38.7 |
| Connecticut | 724 | 687 | 94.8 | 28 | 3.9 |  |  |
| Delaware | 144 | 115 | 80.0 | 28 | 19.5 |  |  |
| Florida | 6,868 | 3,637 | 53.0 | 3,000 | 43.7 |  |  |
| Georgia | 9,900 | 5,279 | 53.3 | 4,485 | 45.3 |  |  |
| Idaho | 8,785 | 347 | 4.0 | 7,335 | 83.5 | 261 | 3.0 |
| Illinois | 1,965 | 1,919 | 97.7 | 32 | 1.6 |  |  |
| Indiana | 1,932 | 1,876 | 97.1 | 46 | 2.4 |  |  |
| Iowa | 1,164 | 1,130 | 97.1 | 15 | 1.3 |  |  |
| Kansas | 1,005 | 932 | 92.8 | 46 | 4.6 |  |  |
| Kentucky | 5,008 | 4,817 | 96.2 | 178 | 3.6 |  |  |
| Louisiana | 6,071 | 3,541 | 58.3 | 2,461 | 40.5 |  |  |
| Maine | 7,083 | 4,132 | 58.3 | 2,934 | 41.4 |  |  |
| Maryland | 988 | 814 | 82.3 | 169 | 17.1 |  |  |
| Massachusetts | 1,218 | 1,001 | 82.2 | 210 | 17.2 |  |  |
| Michigan | 8,161 | 6,073 | 74.4 | 2,019 | 24.7 |  |  |
| Minnesota | 7,153 | 4,890 | 68.4 | 2,183 | 30.5 |  |  |
| Mississippi | 7,772 | 3,999 | 51.5 | 3,628 | 46.7 |  |  |
| Missouri | 6,187 | 5,905 | 95.4 | 257 | 4.1 |  |  |
| Montana | 10,469 | 325 | 3.1 | 8,717 | 83.3 | 576 | 5.5 |
| Nebraska | 567 | 354 | 62.3 | 164 | 28.9 | 4 | 0.7 |
| Nevada | 4,293 | 101 | 2.4 | 169 | 3.9 | 3,721 | 86.7 |
| New Hampshire | 1,899 | 1,470 | 77.4 | 423 | 22.3 |  |  |
| New Jersey | 802 | 591 | 73.7 | 201 | 25.0 |  |  |
| New Mexico | 9,965 | 213 | 2.1 | 1,801 | 18.1 | 7,309 | 73.3 |
| New York | 7,536 | 6,569 | 87.2 | 884 | 11.7 |  |  |
| North Carolina | 7,588 | 4,876 | 64.3 | 2,642 | 34.8 |  |  |
| North Dakota | 327 | 276 | 84.3 | 2 | 0.6 | 42 | 12.8 |
| Ohio | 3,180 | 3,071 | 96.6 | 83 | 2.6 |  |  |
| Oklahoma | 4,834 | 3,753 | 77.6 | 712 | 14.7 | 198 | 4.1 |
| Oklahoma (Great Plains) | 2,575 | 2,051 | 79.7 | 190 | 7.4 | 198 | 7.7 |
| Oklahoma (South Central) | 2,259 | 1,702 | 75.3 | 523 | 23.1 |  |  |
| Oregon | 11,966 | 1,181 | 9.9 | 10,295 | 86.0 | 12 | 0.1 |
| Oregon (West) | 6,167 | 1,069 | 19.3 | 4,947 | 80.2 |  |  |
| Oregon (East) | 5,799 | 112 | 1.9 | 5,348 | 92.2 | 12 | 0.2 |
| Pennsylvania | 6,780 | 6,476 | 95.5 | 258 | 3.8 |  |  |
| Rhode Island | 148 | 129 | 87.2 | 18 | 11.9 |  |  |
| South Carolina | 5,203 | 2,659 | 51.1 | 2,489 | 47.8 |  |  |
| South Dakota | 768 | 188 | 24.5 | 473 | 61.6 | 29 | 3.8 |
| Tennessee | 5,618 | 5,009 | 89.2 | 593 | 10.6 |  |  |
| Texas | 25,050 | 8,035 | 32.1 | 2,396 | 9.6 | 12,933 | 51.7 |
| Texas (Great Plains) | 20,184 | 5,543 | 27.5 | 95 | 0.5 | 12,933 | 64.1 |
| Texas (South Central) | 4,901 | 2,574 | 52.5 | 2,257 | 46.1 |  |  |
| Utah | 7,285 | 659 | 9.0 | 1,156 | 15.9 | 5,204 | 71.4 |
| Vermont | 1,830 | 1,535 | 83.9 | 293 | 16.0 |  |  |
| Virginia | 6,502 | 5,117 | 78.7 | 1,351 | 20.8 |  |  |
| Washington | 8,945 | 884 | 9.9 | 7,654 | 85.6 |  |  |
| Washington (West) | 4,868 | 699 | 14.4 | 4,066 | 83.5 |  |  |
| Washington (East) | 4,077 | 185 | 4.5 | 3,589 | 88.0 |  |  |
| West Virginia | 4,850 | 4,715 | 97.2 | 123 | 2.5 |  |  |
| Wisconsin | 6,863 | 5,516 | 80.4 | 1,284 | 18.7 |  |  |
| Wyoming | 4,239 | 356 | 8.4 | 3,111 | 73.4 | 401 | 9.5 |
