# Supplementary material for: Current aboveground live tree carbon stocks and annual net change in forests of conterminous United States
Source: Carbon Balance Manag. 2021 May 20;16:17. doi: 10.1186/s13021-021-00179-2 (PMC8138985; doi:10.1186/s13021-021-00179-2)
Supplement: Supplementary file 5 — Additional file 5: Table S5. Rates of average annual (aboveground live tree) change by state as calculated from two approaches. [file 13021_2021_179_MOESM5_ESM.docx]

Table S5. Difference in mean average annual change of live aboveground tree carbon by state when calculated by two different approaches: a subset of remeasured forest inventory plots (column 2, from Table S2), or whole-state stock change based on two full inventory cycles (column 3, following Smith et al. 2010). Net annual change in forest area from the stock change approach is also included. Wyoming as well as the Great Plains portions of Oklahoma and Texas are not included because of a lack of remeasured plots.

| **State** | **Remeasured plot difference** | **Whole inventory-cycle difference** | **Net change in forest land** |
| --- | --- | --- | --- |
|  | (tC/ha/y) | (tC/ha/y) | (1000/ha/y) |
| Alabama | 0.94 | 1.02 | 12.22 |
| Arizona | -0.01 | -0.06 | -7.35 |
| Arkansas | 0.76 | 0.73 | 9.60 |
| California | 0.58 | 0.47 | -60.32 |
| Colorado | -0.47 | -0.43 | 5.59 |
| Connecticut | 0.86 | 1.24 | 3.13 |
| Delaware | 0.27 | 0.76 | 0.41 |
| Florida | 0.73 | 0.65 | 2.70 |
| Georgia | 0.84 | 0.55 | -13.78 |
| Idaho | 0.12 | -0.07 | 20.93 |
| Illinois | 0.51 | 0.40 | -0.40 |
| Indiana | 0.51 | 0.65 | 2.84 |
| Iowa | 0.55 | 0.39 | -7.72 |
| Kansas | 0.57 | 0.58 | 10.98 |
| Kentucky | 0.61 | 0.63 | 0.25 |
| Louisiana | 0.73 | 0.75 | 23.85 |
| Maine | 0.38 | 0.32 | -6.21 |
| Maryland | 0.83 | 0.73 | -2.30 |
| Massachusetts | 0.83 | 0.98 | -0.44 |
| Michigan | 0.38 | 0.43 | 11.55 |
| Minnesota | 0.34 | 0.38 | 19.96 |
| Mississippi | 1.82 | 1.38 | -14.10 |
| Missouri | 0.26 | 0.26 | -7.02 |
| Montana | -0.21 | -0.28 | 18.57 |
| Nebraska | 0.08 | -0.06 | -4.25 |
| Nevada | 0.03 | 0.01 | 2.81 |
| New Hampshire | 0.59 | 0.37 | -5.53 |
| New Jersey | 0.57 | 0.62 | -0.85 |
| New Mexico | -0.24 | -0.11 | -12.39 |
| New York | 0.55 | 0.63 | -15.36 |
| North Carolina | 1.03 | 0.93 | 5.52 |
| North Dakota | 0.31 | 0.31 | 3.11 |
| Ohio | 0.44 | 0.25 | -8.46 |
| Oklahoma (S. Central) | 0.32 | 0.17 | -8.70 |
| Oregon (West) | 1.66 | 1.48 | 1.37 |
| Oregon (East) | 0.47 | 0.33 | -26.10 |
| Pennsylvania | 0.57 | 0.72 | 4.51 |
| Rhode Island | 0.84 | 1.40 | 0.85 |
| South Carolina | 0.87 | 0.68 | -8.36 |
| South Dakota | -0.32 | -0.24 | 0.33 |
| Tennessee | 0.75 | 0.62 | -4.27 |
| Texas (S. Central) | 0.52 | 0.22 | -3.85 |
| Utah | -0.09 | -0.13 | -0.05 |
| Vermont | 0.61 | 0.46 | -4.05 |
| Virginia | 1.28 | 1.06 | 6.13 |
| Washington (West) | 1.66 | 0.90 | -8.40 |
| Washington (East) | 0.21 | -0.09 | -5.97 |
| West Virginia | 0.77 | 0.64 | 0.43 |
| Wisconsin | 0.46 | 0.54 | 9.77 |
